# Genome-Wide Identification, Phylogeny, and Expression Analyses of the 14-3-3 Family Reveal Their Involvement in the Development, Ripening, and Abiotic Stress Response in Banana

**DOI:** 10.3389/fpls.2016.01442

**Published:** 2016-09-22

**Authors:** Meiying Li, Licheng Ren, Biyu Xu, Xiaoliang Yang, Qiyu Xia, Pingping He, Susheng Xiao, Anping Guo, Wei Hu, Zhiqiang Jin

**Affiliations:** ^1^Key Laboratory of Biology and Genetic Resources of Tropical Crops, Institute of Tropical Bioscience and Biotechnology, Chinese Academy of Tropical Agricultural SciencesHaikou, China; ^2^Department of Biology, Hainan Medical CollegeHaikou, China; ^3^Key Laboratory of Genetic Improvement of Bananas, Hainan province, Haikou Experimental Station, Chinese Academy of Tropical Agricultural SciencesHaikou, China

**Keywords:** abiotic stress, banana, gene expression, genome-wide, identification, 14-3-3 proteins

## Abstract

Plant 14-3-3 proteins act as critical components of various cellular signaling processes and play an important role in regulating multiple physiological processes. However, less information is known about the 14-3-3 gene family in banana. In this study, 25 14-3-3 genes were identified from the banana genome. Based on the evolutionary analysis, banana 14-3-3 proteins were clustered into ε and non-ε groups. Conserved motif analysis showed that all identified banana 14-3-3 genes had the typical 14-3-3 motif. The gene structure of banana 14-3-3 genes showed distinct class-specific divergence between the ε group and the non-ε group. Most banana 14-3-3 genes showed strong transcript accumulation changes during fruit development and postharvest ripening in two banana varieties, indicating that they might be involved in regulating fruit development and ripening. Moreover, some 14-3-3 genes also showed great changes after osmotic, cold, and salt treatments in two banana varieties, suggested their potential role in regulating banana response to abiotic stress. Taken together, this systemic analysis reveals the involvement of banana 14-3-3 genes in fruit development, postharvest ripening, and response to abiotic stress and provides useful information for understanding the functions of 14-3-3 genes in banana.

## Introduction

14-3-3 proteins are highly conserved regulatory molecules existing in virtually all multicellular eukaryotic tissues. They have been recognized as the best characterized and most important phosphopeptide-binding proteins (Sehnke et al., 2002; Chevalier et al., 2009; Paul et al., 2012; Cotelle and Leonhardt, 2016). In the model plant *Arabidopsis*, 14-3-3 proteins were named as general regulatory factor (GRF) with an Arabic number (Chevalier et al., 2009). Generally, 14-3-3 proteins regulate phosphorylated clients through the specific groove structures formed by their homo- or hetero-dimers (Chevalier et al., 2009; de Boer et al., 2013; Cotelle and Leonhardt, 2016). The interesting properties of the dimer groove structures make 14-3-3 proteins function as scaffolding proteins, which can bind to a wide range of target proteins (Chevalier et al., 2009; de Boer et al., 2013), such as carbon and nitrogen metabolic enzymes (Comparot et al., 2003; Diaz et al., 2011), key point enzymes associated with ion pumps and channels (Bunney et al., 2002; Kanczewska et al., 2005; Ottmann et al., 2007; Latz et al., 2013; Chen et al., 2015), various protein kinases, transcription factors and proteins, or transcription factors associated with hormone signal transduction (Schoonheim et al., 2007; Jaspert et al., 2011; Ho et al., 2013; Latz et al., 2013; de Boer et al., 2013; Ito et al., 2014; Kawamoto et al., 2015). The interaction process between 14-3-3 proteins and their different clients lead to various biochemical changes in cellular processes, including the stability, activity, degradation, intracellular localization, and the binding ability with other client proteins of the protein or enzyme (Chevalier et al., 2009; Paul et al., 2012; de Boer et al., 2013; Cotelle and Leonhardt, 2016).

Since the first plant 14-3-3 isoform was isolated from maize (de Vetten et al., 1992), genome-wide analyses have identified 13 14-3-3s from *Arabidopsis* (DeLille et al., 2001; Rosenquist et al., 2001; Chevalier et al., 2009), 8 from rice (Chen et al., 2006; Yao et al., 2007), 31 from cotton (Sun et al., 2011), 9 from common bean (Tian et al., 2015), 18 from soybean (Li and Dhaubhadel, 2011), 14 from *Populus trichocarpa* (Li et al., 2015), 12 from tomato (Xu and Shi, 2006), and 21 from *Brassica rapa* (Chandna et al., 2016). Biochemical and genetic analyses have revealed that the function of 14-3-3 proteins is related to plant growth and development. Multiple mutant analysis indicated that *Arabidopsis* 14-3-3 proteins regulate root growth (van Kleeff et al., 2014; He et al., 2015), chloroplast division, photosynthesis, and leaf longevity (Vercruyssen et al., 2015). *GsGF14o* from glycine soja was reported to participate in stomatal and root hair development (Sun et al., 2014). Overexpression of *Gh14-3-3L* in cotton promoted fiber elongation and maturation (Zhou et al., 2015). In addition, expression analyses of the 14-3-3 gene family in various species indicated that its expression is altered under various abiotic stress, such as drought, salt, or cold (Li and Dhaubhadel, 2011; Kumar et al., 2015; Li et al., 2015; Tian et al., 2015; Chandna et al., 2016). Further studies support that 14-3-3 genes play a role in plant response to abiotic stress. Overexpression of the *Arabidopsis* 14-3-3 protein *GF14 lambda* in cotton could lead to a “stay-green” phenotype and improved stress tolerance (Yan et al., 2004). *Arabidopsis* 14-3-3 lambda and kappa were identified as important regulators of salt tolerance (Tseng et al., 2012; Zhou et al., 2014) while 14-3-3 psi was involved in freezing tolerance and cold acclimation (Catalá et al., 2014). Taken together, these studies have shown that the plant 14-3-3 gene family is involved in regulating plant growth, development, and response to various stresses.

As a large annual monocotyledonous herbaceous plant, banana (*Musa acuminata* L.) is one of the most popular fresh fruit. Banana fruit quality is determined by development and postharvest ripening processes and it plays an important role in the commodity economy (Raza et al., 2016). Also, banana production is often threatened by various environmental stresses such as low temperature, drought, salt damage, and various diseases (Ravi et al., 2013; Yan et al., 2015; Yang et al., 2015; Raza et al., 2016). Thus, it is necessary to study the molecular mechanism underlying banana fruit development, postharvest ripening, and response to various abiotic stresses. Considering the importance of 14-3-3 proteins in regulating plant growth, development, as well as responses to abiotic stresses, a comprehensive analysis of banana 14-3-3 genes was conducted. In the study, a total of 25 14-3-3s genes were identified in banana. Further, their phylogenic relationship, gene structures, and protein motifs were studied in detail. Finally, the expression of banana 14-3-3 genes in various organs, different phases of fruit development and ripening, and responses to various stresses in Ba Xi Jiao (*Musa acuminate* L. AAA group cv. Cavendish, BX) and Fen Jiao (*Musa* ABB Pisang Awak, FJ) varieties were comprehensively characterized. The detailed characterization of the banana 14-3-3 gene family provides a foundation and useful genetic resources for further functional characterization of potential targets of 14-3-3 and the genetic improvement of bananas.

## Materials and methods

### Plant materials and treatments

In this study, 2 banana cultivated varieties, BX and FJ, were selected because FJ ripened faster and is more tolerant to abiotic stress than BX. The distinct characteristics between the two varieties are benefit for performing comparative analyses of the underlying mechanism. The seedlings of these 2 varieties at the 5-leaf growth stage were obtained from the banana tissue culture center (Institute of Banana and Plantain, Chinese Academy of Tropical Agricultural Sciences, Danzhou, China). Then, plants of each variety was placed in a growth chamber (28°C; 200 μmol·m^−2^·s^−1^ light intensity; 16-h light/8-h dark cycle; 70% RH), the others were planted in planting base (Institute of Tropical Bioscience and Biotechnology, Chinese Academy of Tropical Agricultural Sciences, Wenchang, China).

### Identification and phylogenetic analyses of the 14-3-3 gene family in banana

Banana 14-3-3 genes were obtained from the DH-Pahang (*Musa acuminate*, A-genome, 2*n* = 22) genome database (D'Hont et al., 2012). The known 14-3-3s was used to search the 14-3-3 proteins from the banana genome sequence using a hidden markov model (protein domain code: IPR000308; Finn et al., 2011). To further identify possible 14-3-3s in the banana database, BLAST analyses were performed to further query with all 14-3-3s from *Arabidopsis* and rice. To further validate all identified banana 14-3-3 genes, a conserved domain search was conducted by using the CDD and PFAM databases. Then, the identities of banana 14-3-3s were analyzed based on multiple alignment by Vector NIT Suite 11.0. Amino acid sequences and genome sequences of 14-3-3s in *Arabidopsis* and rice were obtained from RGAP, and UniPort databases, respectively. A bootstrap neighbor-joining phylogenetic tree with the identified 25 14-3-3s from banana and all 14-3-3s from *Arabidopsis* and rice was constructed by using Clustal X 2.0 and MEGA 5.0 based on their amino acid sequences (Larkin et al., 2007; Tamura et al., 2011).

### Characterization of protein properties and sequences

The relative molecular weights (RMW) and isoelectric points (pI) of the identified banana 14-3-3 proteins were predicted by the ExPASy proteomics server database (http://expasy.org/). Banana 14-3-3 protein motifs were analyzed with MEME software (http://meme-suite.org/tools/meme). The optimum width of motifs ranged from 8 to 50 with the maximum number of motifs being 10. The predicted motifs of banana 14-3-3 proteins were further annotated with an InterProScan database search (http://www.ebi.ac.uk/Tools/pfa/iprscan/). Gene structures of banana 14-3-3 genes were determined with GSDS software (http://gsds.cbi.pku.edu.cn/) based on their genome and coding sequences.

### Transcriptome analysis

To investigate the transcriptional accumulation of 14-3-3 genes in different organs of the BX and FJ varieties, roots, leaves, and fruits at different developmental stages and during postharvest ripening stages were collected as follows. Roots, leaves, and fruits at 80 days after flowering (DAF) were sampled for expression analysis of 14-3-3 genes in different tissues. Banana fruits at distinct development stages, i.e., 0 DAF, 20 DAF, and 80 DAF, were collected to assess the expression patterns of banana 14-3-3s during fruit development. As a previous reports, postharvest ripening processes of banana fruit were classified into FG (full green), TY (trace yellow), MG (more green than yellow), MY (more yellow than green), GT (green tip), FY (full yellow), and YB (yellow flecked with brown spots) stages (Pua et al., 2003). In this study, to examine the expression of 14-3-3 genes during fruit postharvest ripening processes, fruits at 8 and 14 days DPH in BX and at 3 and 6 DPH in FJ were collected. The 5-leaf stage banana seedlings were treated with 200 mM mannitol for 7 days, 300 mM NaCl for 7 days, or low temperature (4°C) for 22 h, respectively. All leaf samples were collected and frozen quickly in liquid nitrogen and stored at −80°C for RNA extraction. The global expression patterns of banana genes were determined by using RNA-seq. Total RNA was extracted using a plant RNA extraction kit (Tiangen, China) and was converted into cDNA by using a RevertAid First Strand cDNA Synthesis kit (Ferments). The cDNA libraries were constructed based on Illumina protocols and subsequently subjected to sequencing by Illumina GAII following the Illumina RNA sequencing protocol. Each sample contained 2 biological replicates. The sequencing depth was 5.34X on average and the coverage was more than 95%. FASTX-toolkit was used to remove the adapter sequences. Clean reads were produced by removing low quality sequences and examining the sequence quality using FastQC. Clean reads were mapped to the DH-Pahang genome (*Musa acuminate*, A-genome, 2*n* = 22) with Tophat v.2.0.10. Cufinks was employed to carry out the transcriptome assemblies. Gene expression levels were calculated as FPKM. Differentially expressed genes were identified using DEGseq. The heat-map was constructed with MeV 4.9 and Java Treeview softwares according to the manufacturer's protocol.

### qRT-PCR analysis

Expression of MaGRF genes were detected by quantitative rea-time polymerase chain reaction (qRT-PCR) analysis using SYBR® Premix Ex Taq™(TaKaRa, Shiga, Japan) chemistry on a StepOnePlus Real-Time PCR (Applied Biosystems, Foster City, CA) instrument. Primer pairs that had high specificity and efficiency were selected to conduct quantification assay (Table S6). The banana MaUBQ2 (HQ853254) was selected as internal control to normalize the relative expression of target genes (Chen et al., 2011). The relative expression levels of the target genes were assessed based on 2^−ΔΔCt^ method. Each sample contains three replicates.

## Results

### Identification and phylogenetic analysis of banana 14-3-3 genes

**Figure 1:**
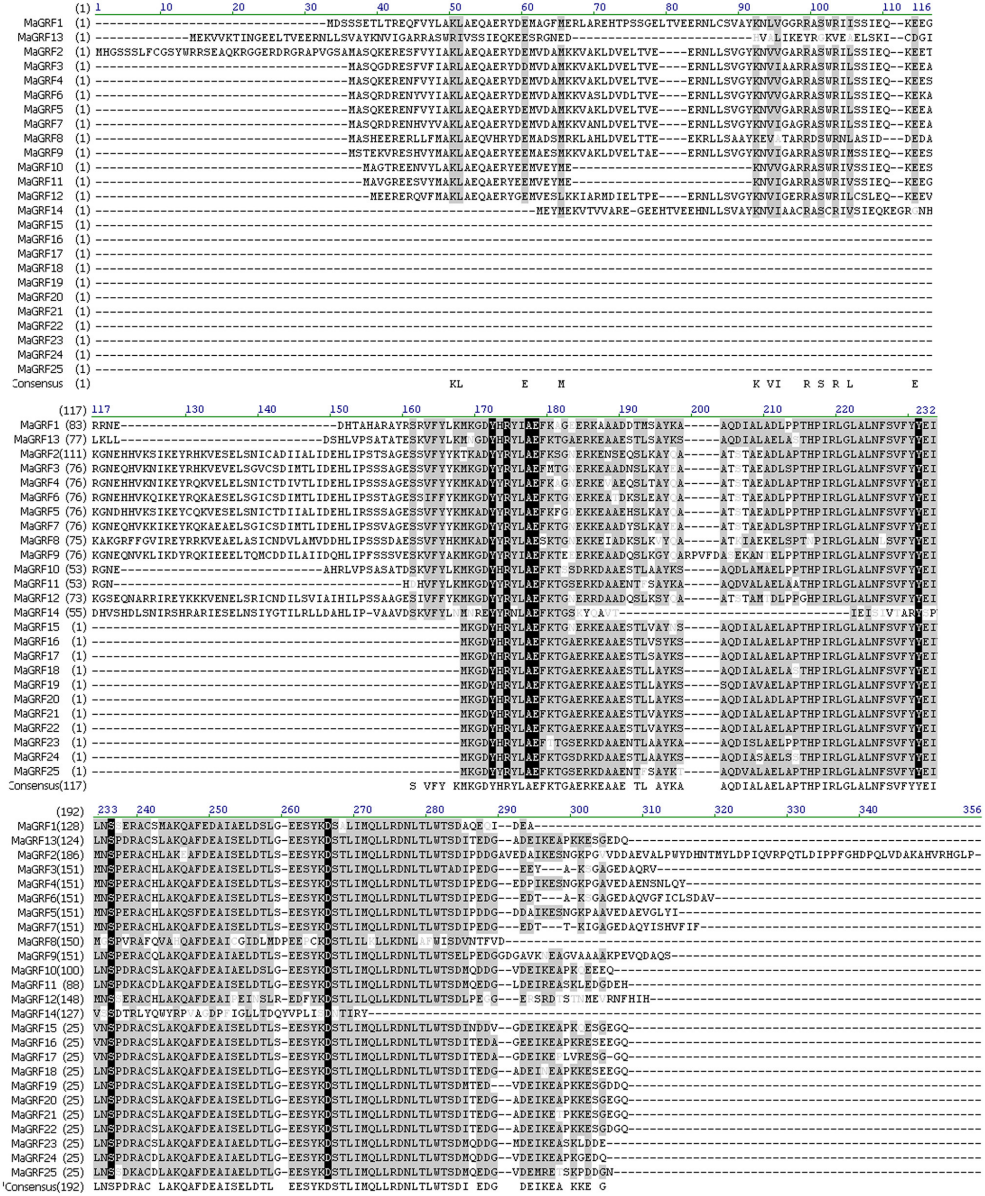
**Amino acid alignment of the deduced banana 14-3-3 proteins**. Multiple alignments of the amino acid sequences of 25 banana 14-3-3 genes were analyzed by Vector NIT Suite 11.0, amino acids with high identities were shown in black or gray background.

BLAST and the hidden markov model were conducted to identify all banana 14-3-3 genes with *Arabidopsis* and rice 14-3-3 sequences as queries. A total of 25 non-redundant 14-3-3 (designed as *MaGRF1* to *25*) genes were identified in the banana genome, which was supported by conserved domain and multiple sequence alignment analyses (Figure 1, Table S1). The 25 predicated banana 14-3-3 proteins ranged from 130 (*MaGRF23, MaGRF24*) to 344 (*MaGRF2*) amino acid residues in length, and their relative molecular mass varied from 14.529 KDa (*MaGRF24*) to 38.789 KDa (*MaGRF2*), with the pIs in the range of 4.31–6.41, suggesting their potentially different roles in regulating cellular processes under different environments (Figure 1, Table S2).

**Figure 2:**
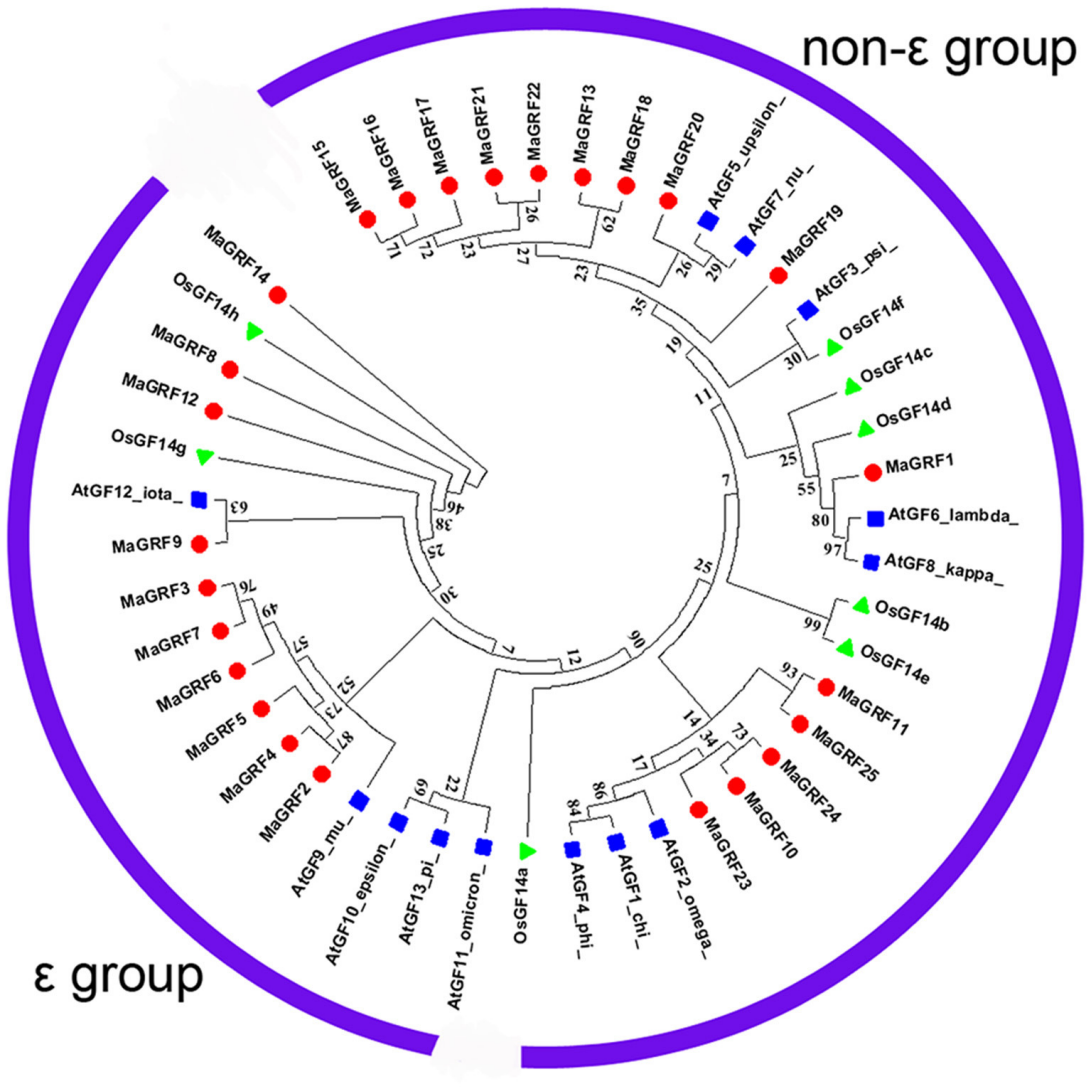
**Phylogenetic analysis of 14-3-3s from *Arabidopsis*, rice, and banana using Neighbor-joining method**. The Phylogenetic tree was constructed by using ClustalX 2.0 and MEGA5.0 software with pair-wise deletion option. Tree reliability was assessed using 1000 bootstraps replicates. The numbers indicated for each clade represent bootstrap support values given as percentages. Two groups were shown as ε group and the non-ε group. Solid round, 25 14-3-3 proteins from banana; Solid Square, 12 14-3-3 proteins from *Arabidopsis*; Solid triangle, 8 14-3-3 proteins from rice.

Based on the alignments of 14-3-3 proteins from banana, rice, and *Arabidopsis*, a phylogenetic tree was constructed to understand their evolutionary relationships (Figure 2). The results showed that all identified 14-3-3 proteins from banana were clearly classified into the ε- and the non-ε group. According to the phylogenetic relationship, 10 banana 14-3-3 genes (*MaGRF2, 6, 3, 7, 8, 9, 12, 5, 4*, and *14*) together with *Arabidopsis AtGF9, 10, 13, 11, 12*, and the rice *OsGF14g, h* were classified into the ε group. The other 15 banana 14-3-3 genes, i.e., *MaGRF1, 11, 10, 13*, and *MaGRF15-25* together with *Arabidopsis AtGF6, 8, 7, 3, 5, 4, 1*, and rice *OsGF14a, c, e, b, f*, *d*, belonged to the non-ε group.

### Gene structure and conserved motifs of 14-3-3 genes in banana

**Figure 3:**
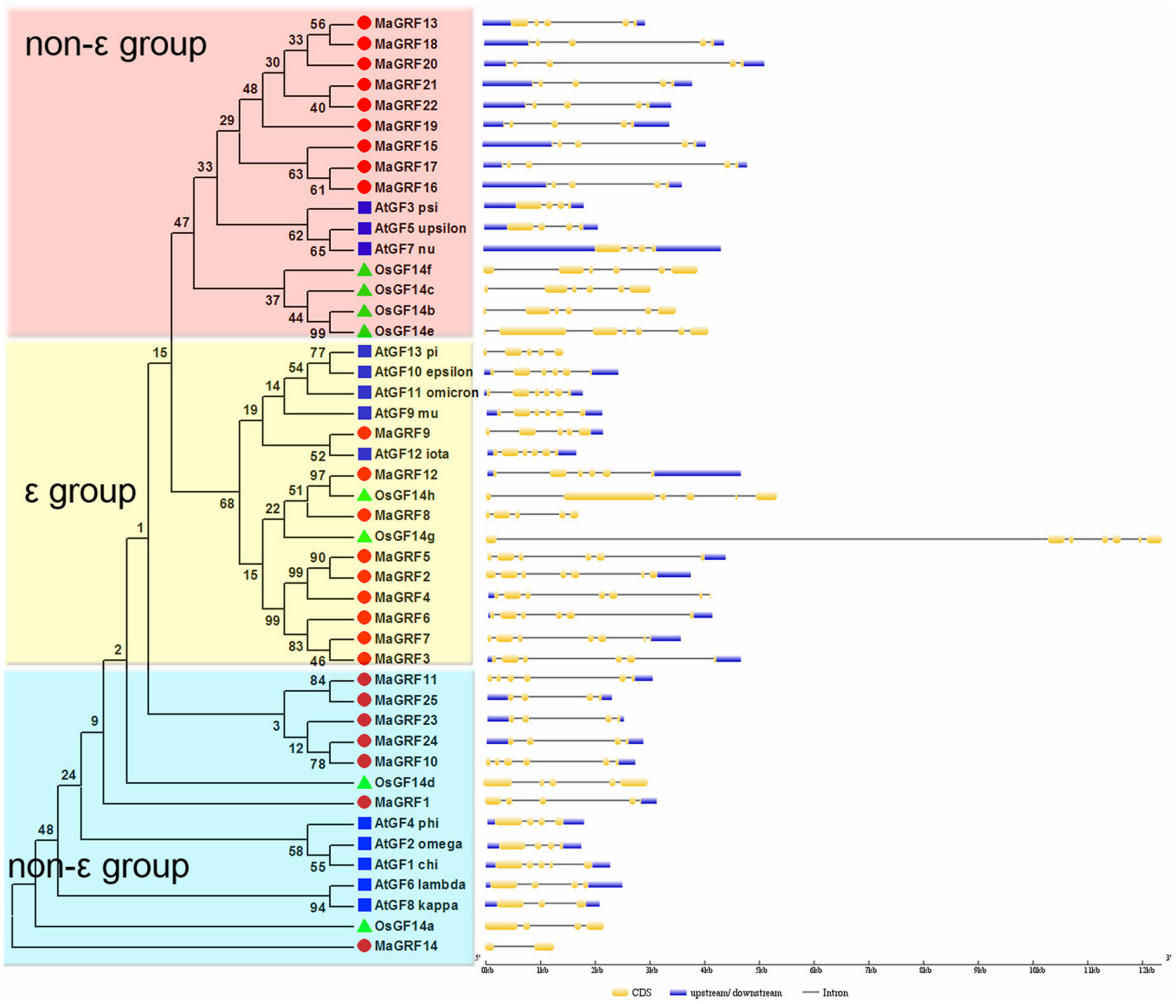
**The Phylogenetic relationship and exon-intron structure analyses of 14-3-3s from banana, *Arabidopsis*, and rice**. The neighbor-joining tree was created using ClustalX 2.0 and MEGA5.0 with amino acid sequences of 14-3-3s from banana, *Arabidopsis* and rice. Two groups are indicated with different color backgrounds. Exon-intron structure analyses were conducted using the GSDS database. Lengths of exons and introns of each *14-3-3* gene were exhibited proportionally.

Exon-intron structural divergence within families plays a key role in the evolution of gene families. To understand the structural diversity of banana 14-3-3 genes, exon-intron organization among the coding sequence of 46 14-3-3s (25 from banana, 13 from *Arabidopsis* and 8 from rice) was conducted using the Gene Structure Display Server (GSDS, http://gsds.cbi.pku.edu.cn/) based on an evolutionary analysis. The results showed that banana 14-3-3 genes contained 2-7 exons (Figure 3). Non-ε group banana 14-3-3 genes contained 2-6 exons, whereas ε group genes had 5–7 exons. The exon-intron organization was different between the ε group and the non-ε group 14-3-3 genes of the 3 species, indicating the diversity of expansion and evolution between the ε group and the non-ε group plant 14-3-3 genes (Figure 3).

**Figure 4:**
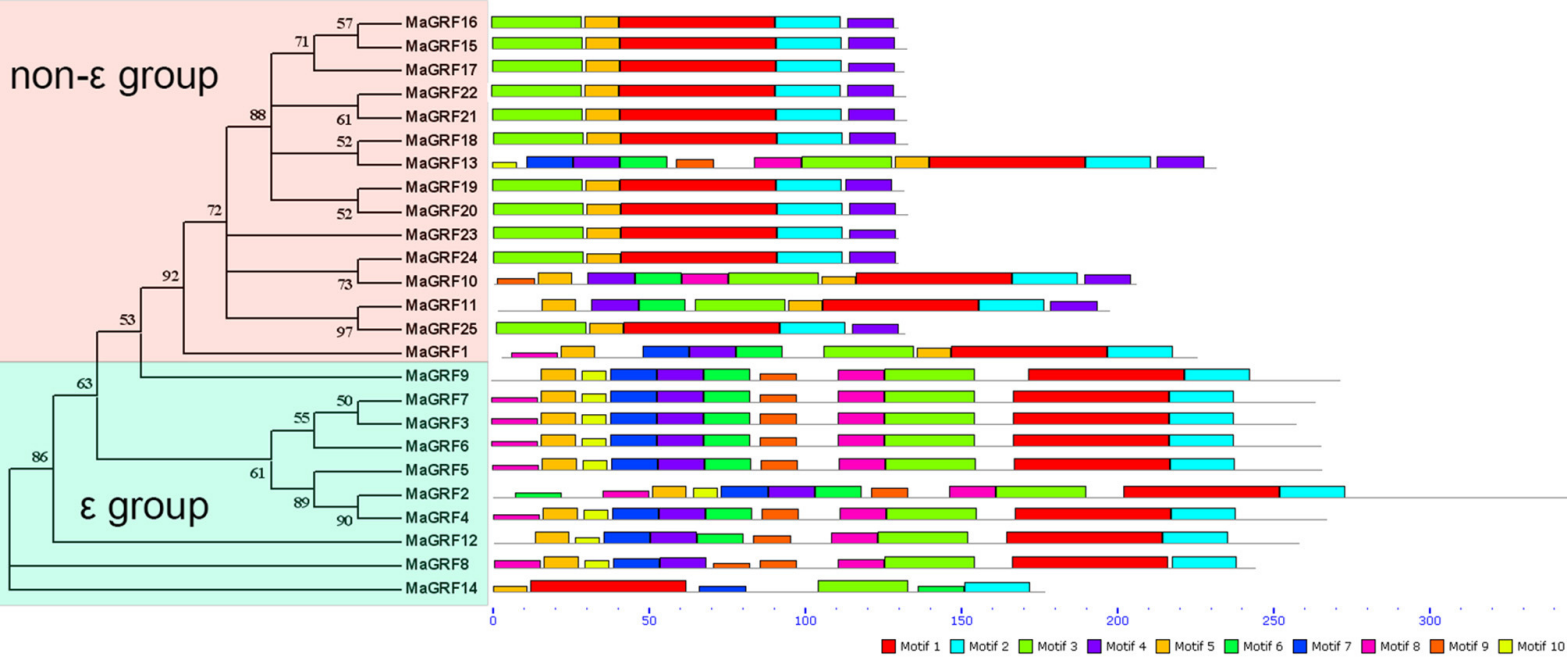
**The phylogenetic relationship and motif analyses of banana 14-3-3s**. The neighbor-joining tree was created using ClustalX 2.0 and MEGA5.0 with amino acid sequences of banana 14-3-3s. Two groups are indicated with different color backgrounds. All motifs were identified by the MEME database with complete amino acid sequences of banana 14-3-3s. Lengths of motifs for each banana 14-3-3 protein were exhibited proportionally.

To explore the structural diversity and predict the function of banana 14-3-3 proteins, a total of 10 conserved motifs in banana 14-3-3 genes were identified by MEME software and further annotated with InterPro Scan 5 (Figure 4, Figure S1). The results suggested that 6 motifs (motifs 1–6) were annotated as 14-3-3 protein domains, which are basic characteristics of the 14-3-3 gene family. According to the motif analysis, all identified banana 14-3-3 proteins contained the typical 14-3-3 domain motifs. All ε group banana 14-3-3 proteins contain the motifs 1, 2, 3, 4, 5, whereas all non-ε group banana 14-3-3 proteins share the motifs 1, 2, 3, 5, 7. Most ε group banana 14-3-3 proteins had motif 3 and motif 4 at the N terminal and C terminal, respectively. In contrast, most non-ε group banana 14-3-3 proteins showed motif 8 and motif 2 at the N terminal and C terminal, respectively. The motif structure conservation and divergence might indicate their group and function specific to banana 14-3-3s.

### Expression analysis of 14-3-3 genes in different tissues of two banana varieties

**Figure 5:**
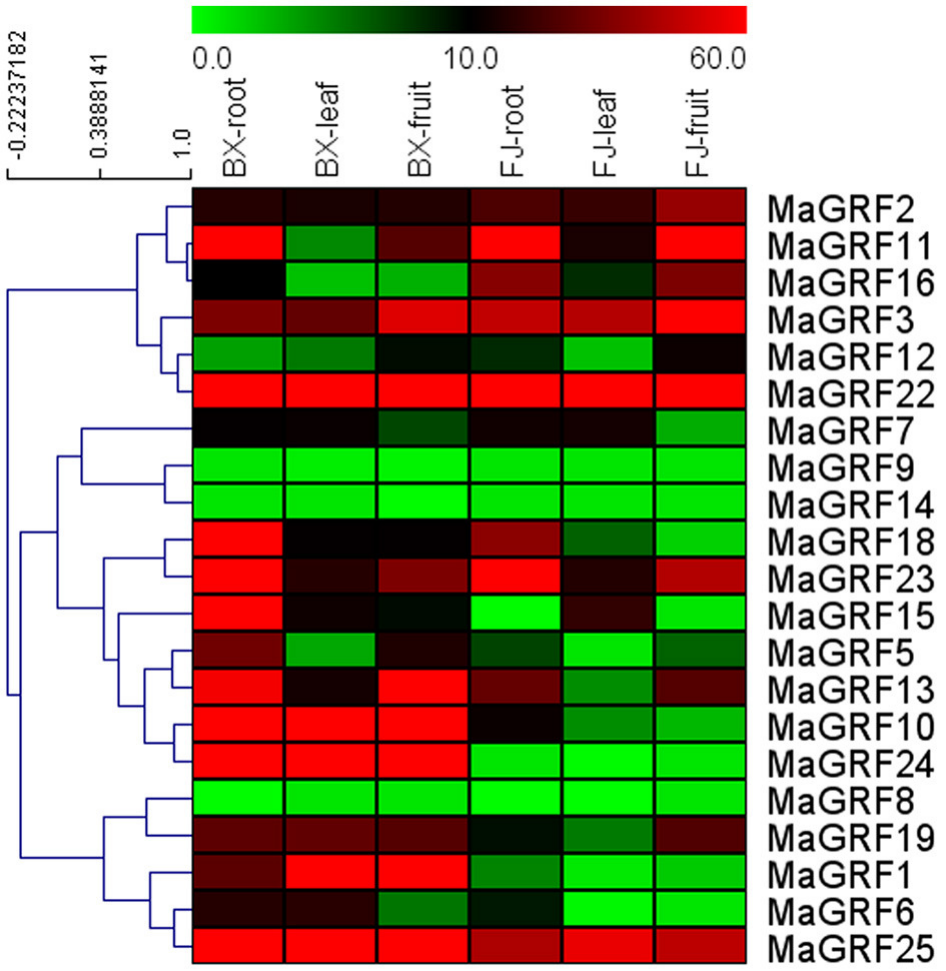
**Expression analysis of banana 14-3-3 genes in roots, leaves, and fruits of two cultivated banana varieties**. Banana leaves and roots at the five-leaf stage and fruits at 80 DAF in the BX and FJ varieties were sampled to detect the expression profiles of 14-3-3s in various tissues. The heat-map was created based on the transcriptomic data of 14-3-3s from two independent experiments. The scale represents relative signal intensity values.

To investigate the role of 14-3-3 genes in banana growth and development, expression patterns of 14-3-3 genes in different organs, including roots, leaves, and fruits, were tested in 2 cultivated banana varieties, BX and FJ. The transcripts of 21 banana 14-3-3 genes (except *MaGRF4, 20, 21*, and *17*) in different tissues of 2 varieties were obtained based on transcript data (Figure 5, Table S3).

In BX, 19 (90.5%) genes (except *MaGRF8 and 14*) expressed in all tissues examined, among which 17 (*MaGRF1, 2, 3, 5, 6, 7, 10, 11, 13, 15, 16, 18, 19, 22, 23, 24*, and 25), 14 (*MaGRF1, 2, 3, 6, 7, 10, 13, 15, 18, 19, 22, 23, 24*, and *25*), and 13 (*MaGRF1, 2, 3, 5, 10, 11, 13, 18, 19, 22, 23, 24*, and *25*) genes had high transcriptional abundance (value>10) in roots, leaves, and fruits, respectively. In addition, there were 11 (*MaGRF1, 2, 3, 10, 13, 18, 19, 22, 23, 24*, and *25*) genes with high transcriptional abundance (value>10) in all organs tested.

In FJ, 18 (85.7%), 19 (90.5%), and 15 (71.4%) of them expressed in roots, leaves, and fruits, respectively, in which 11 (*MaGRF2, 3, 7, 10, 11, 13, 16, 18*, 22, *23*, and *25*), 8 (*MaGRF2, 3, 7, 11, 15*, 22, *23*, and *25*), and 10 (*MaGRF2, 3, 11, 12, 13, 16, 19*, 22, *23*, and *25*) genes had high transcriptional abundance (value>10) in roots, leaves, and fruits, respectively. There were 6 genes (*MaGRF2, 3, 11, 22, 23*, and *25*) with high expression levels (value>10) in all tissues examined.

To compare the expression patterns of banana *14-3-3s* in various organs of BX and FJ, 19 (90.5%) genes expressed in all BX organs examined, only 15 (71.4%) of them expressed in all organs of FJ. The number of genes with high expression levels (value>10) was more in BX than in FJ in roots, leaves, and fruits. Some genes showed similar expression profiles in BX and FJ, including *MaGRF11, 3, 12, 22, 7, 9, 14, 18, 23, 13, 8*, and *25*, indicating that these genes had similar functions in regulating organs development of BX and FJ. However, some genes exhibited deferential expression profiles between BX and FJ. For example, *MaGRF10* and *MaGRF24* showed strong expression (value>67) in the 3 tissues of BX, whereas low expression (value < 13) in the 3 tissues of FJ. On the contrary, *MaGRF16* had abundant transcripts (value>34) in roots and fruits of FJ, whereas low transcripts (value < 11) in roots and fruits of BX. This indicates thatsome 14-3-3 genes may play distinct roles for organs development in different banana varieties. Additionally, 5 genes (*MaGRF2, 3, 22, 23*, and *25*) showed high expression levels (value>10) in all tissues of BX and FJ examined, indicating their important roles in regulating organ development. Overall, tissue expression profiles of banana 14-3-3 genes in different varieties may provide insight for future studies on tissue development and function.

### Expression analysis of banana 14-3-3 genes in different stages of fruit development and ripening of two banana varieties

**Figure 6:**
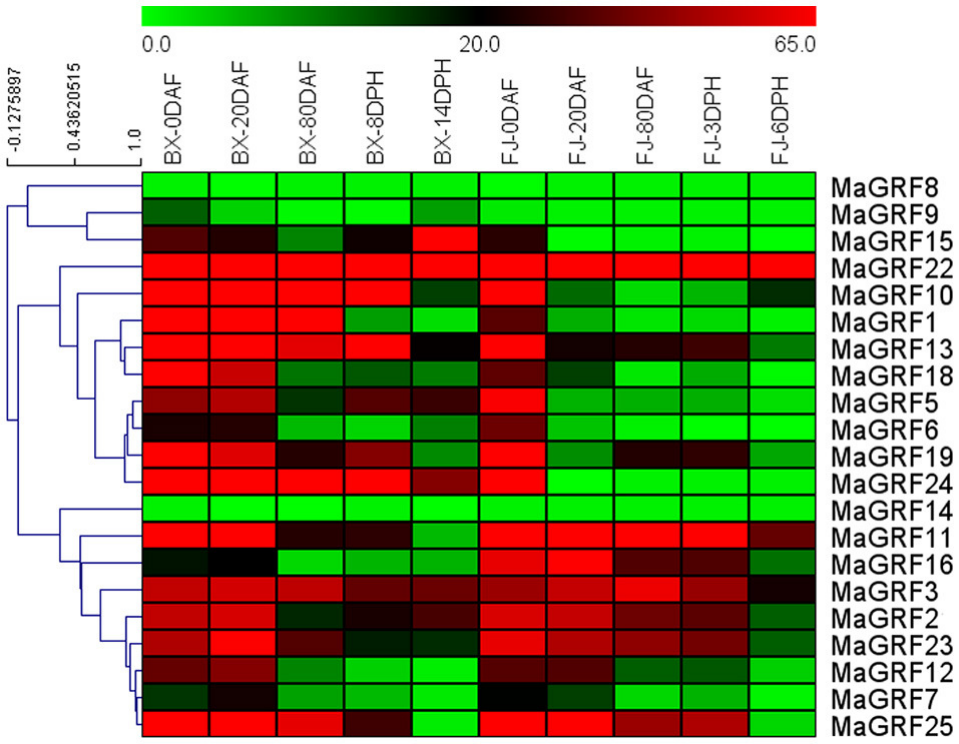
**Expression analysis of 14-3-3s in different stages of fruit development and ripening in two banana varieties**. Fruits of 0, 20, and 80 DAF in BX and FJ varieties were sampled to assess the expression patterns of 14-3-3 genes during fruit development. Fruits at 8 and 14 DPH in BX and at 3 and 6 DPH in FJ were sampled to examine the expression patterns of 14-3-3s during postharvest ripening process. The heat-map was created based on transcriptomic data of 14-3-3s from two independent experiments. The scale represents relative signal intensity values.

To investigate the functions of 14-3-3 genes in regulating banana fruit development and postharvest ripening, changes in banana 14-3-3 genes expression at 0, 20, and 80 DAF in BX and FJ, 8 and 14 DPH in BX, and 3 and 6 DPH in FJ were analyzed according to transcriptomic data. There were 21 14-3-3 genes expressed at different phases of fruit development and postharvest ripening in 2 varieties (Figure 6, Table S4).

In BX, 19 (no *MaGRF8* and *14*), 21, 20 (no *MaGRF8*), 19 (no *MaGRF8* and *14*), and 20 (no *MaGRF8*) 14-3-3 genes expressed at 0, 20, 80 DAF, as well as 8 and 14 DPH, respectively. There were 15 (71.4%), 14 (66.67%), 8 (*MaGRF1, 3, 10, 13, 22, 23, 24*, and *25*), 8 (*MaGRF3, 5, 10, 13, 19, 22, 24* and *25*), and 6 (*MaGRF2, 3, 5, 15, 22* and *24*) banana 14-3-3 genes with high transcriptional abundance (value>30) at 0, 20, 80 DAF, and 8 and 14 DPH, respectively. Notably, *MaGRF22, 24* and *3* genes had high transcriptional accumulation (value>30) at all phases of fruit development and postharvest ripening.

In FJ, 20 (no *MaGRF14*), 18 (no *MaGRF8, 9, 14*), 16 (no *MaGRF6, 8, 9, 14, 15, 24*), 17 (no *MaGRF8, 9, 14, 15, 24*), and 17 (no *MaGRF1, 8, 9, 14, 24*) 14-3-3 genes had expression at 0, 20, and 80 DAF, as well as 8 and 14 DPH, respectively. Furthermore, 16 (76.2%), 8 (*MaGRF2, 3, 11, 12, 16, 22, 23*, and *25*), 7 (*MaGRF2, 3, 11, 16, 22, 23*, and *25*), 8 (*MaGRF2, 3, 11, 13, 16, 22, 23*, and *25*), and 2 (*MaGRF22* and *11*) genes showed high transcrips (value>30) at 0, 20, 80 DAF, and 3 and 6 DPH, respectively. Notably *MaGRF22* and *11* displayed high transcripts (value>30) at all phases of fruit development and postharvest ripening.

By comparing the expression paterns of banana 14-3-3 genes at distinct phases of fruit development and postharvest ripening in BX and FJ, 19 banana 14-3-3 genes (~90.5%) showed expression at all stages tested in BX, whereas only 14 of them (~66.7%) showed expression at all stages examined in FJ. Similar expression patterns could be observed at 0 DAF in BX and FJ, indicating that banana 14-3-3 genes played a similar role during early fruit development stages of the two varieties. However, 10 genes (*MaGRF1, 5, 6, 9, 10, 13, 15, 19*, and *24*) had higher expression levels at subsequent stages in BX compared to those in FJ, whereas only 2 genes showed higher expression levels in FJ than in BX. These findings imply a significant transcriptional response of *MaGRFs* during BX fruit development and postharvest ripening processes. In addition, *MaGRF22* displayed high transcriptional abundance (value>80) at all phases in BX and FJ.

### Expression analysis of banana 14-3-3 genes responding to cold, salt, and osmotic stresses of two banana varieties

**Figure 7:**
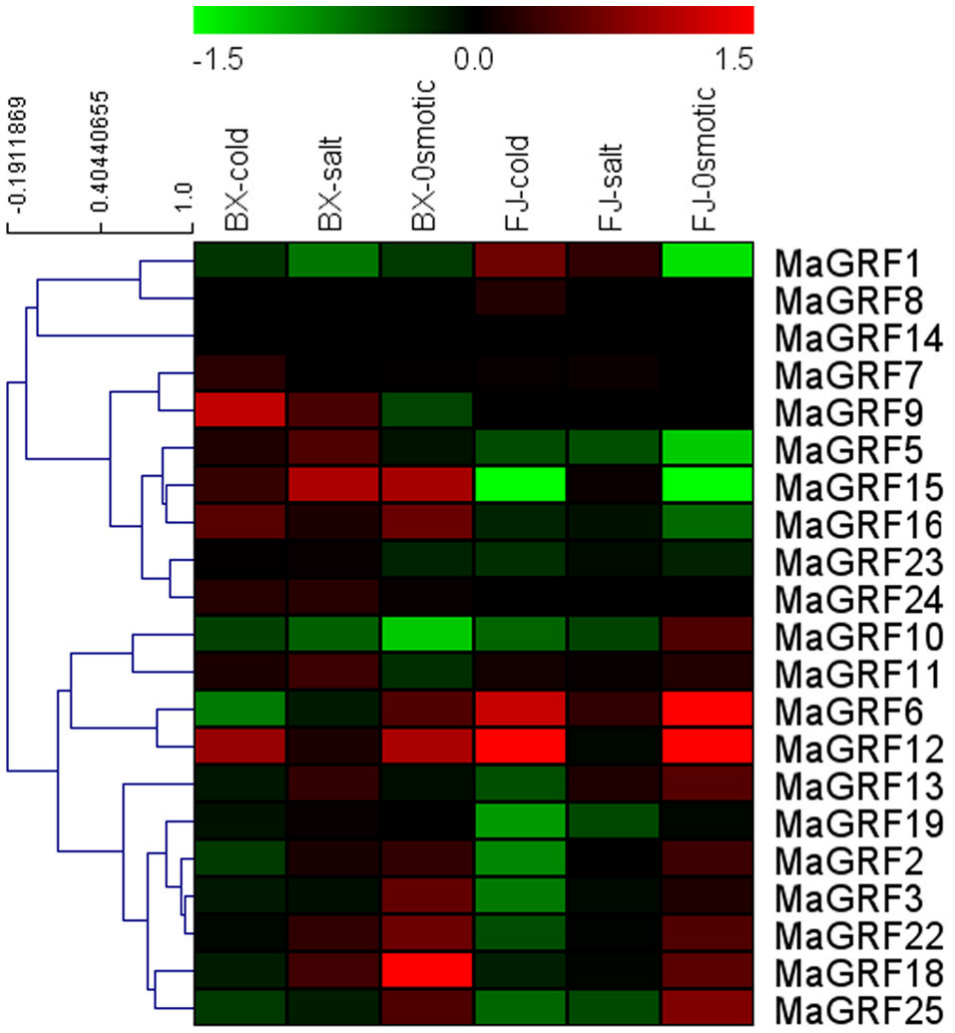
**Expression analysis of 14-3-3s in response to cold, salt, and osmotic treatments in two banana varieties**. For osmotic and salt treatments, banana seedlings at the five-leaf stage grown in soil were irrigated with 200 mM mannitol or 300 mM NaCl for 7 days, respectively. For cold treatment, banana seedlings were cultured in a growth chamber with a temperature that was maintained at 4°C for 22 h. Log_2_-based values were used to create the heat-map based on transcriptomic data of 14-3-3s from two independent experiments. The scale represents relative signal intensity values.

Much evidence had indicated that 14-3-3 genes participated in plant response to various stresses, including drought, cold, and salt. To better understand 14-3-3 genes in response to these 3 stresses, expression of 14-3-3 genes in leaves of BX and FJ was examined under salt, cold, and osmotic treatments. A heat-map representing the expression profile of 21 banana 14-3-3 genes was created using transcriptomic data (Figure 7, Table S5).

For BX, 9 (42.9%), 13 (61.9%), and 12 (57.1%) *MaGRF* genes were upregulated after cold, salt, and osmotic treatments, respectively, whereas 10 (47.6%), 6 (28.5%), and 7 (33.3%) banana 14-3-3 genes were downregulated under the treatment of cold, salt, and osmotic, respectively. Expression of *MaGRF9, 15*, and *18* were strongly upregulated (value>1) by cold, salt, and osmotic stress, respectively. Four genes (*MaGRF12, 16, 15*, and *24*) were upregulated after each of the stress treatments, and 2 genes (*MaGRF1* and *10*) were downregulated after each of the stress treatments.

In FJ, 6 (*MaGRF1, 6, 7, 8, 11*, and 12), 7 (*MaGRF1, 2, 6, 7, 11, 13*, and *15*), and 11 (*MaGRF2, 3, 6, 7, 10, 11, 12, 13, 18, 22*, and *25*) banana 14-3-3 genes showed induction under cold, salt, and osmotic treatments, respectively. Conversely, expression of 57.1% (12/21), 47.6% (10/21), and 28.6% (6/21) banana 14-3-3 genes were inhibited after cold, salt, and osmotic stress, respectively. *MaGRF6* and *12* were significantly induced (value>1) by cold and osmotic treatments.

Overall, the number of upregulated banana 14-3-3 genes by cold, salt, and osmotic stress was more in BX than that in FJ. Three genes (*MaGRF2, 3*, and *25*) presented similar expression patterns after salt, cold, and osmotic treatments, respectively, indicating that their similar functions in BX and FJ under the cold, salt, and osmotic treatments. However, there were 6 (*MaGRF1, 5, 6, 15, 16*, and *23*), 10 (*MaGRF1, 5, 6, 7, 12, 16, 18, 19, 22*, and *23*), and 6 (*MaGRF10, 11, 13, 15, 16*, and *19*) genes showing differential expression patterns between BX and FJ under cold, salt, and osmotic treatments, respectively. Notably, *MaGRF24* was induced after cold, salt, and osmotic stress in BX, but it has no detection expression after these three treatments in FJ.

### Validation of the differentially expressed 14-3-3 genes by qRT-PCR analysis

According to the RNA-seq data, MaGRF3, MaGRF16, and MaGRF25 showed high expression levels in different organs, or abundant transcripts during most stages of fruit development and ripening, or upregulation after abiotic stress treatments in BX or FJ. These differentially expressed MaGRF genes were selected for qRT-PCR analysis to validate the RNA-seq data. After normalization, the majority of selected MaGRF genes, except for MaGRF16 in BX 14 DPH, FJ fruits, and FJ 80 DAF, and MaGRF25 in BX leaves and FJ roots, had the same trend and consistent results between transcriptomic data and qRT-PCR data (Figure S2). These results indicate that transcriptomic data are suitable for analyzing the expression patterns of 14-3-3 genes in two banana varieties.

## Discussion

Bananas are an important and popular fresh fruit worldwide and play a key role in the economy of tropical and subtropical areas as a food commodity. To manipulate banana breeding, it is essential to explore the mechanisms underlying banana fruit development, postharvest ripening, and responses to abiotic stresses. Plant 14-3-3 proteins play key roles in regulating plant growth, development, and the response to abiotic stresses and are considered crucial mediators in many biological and physiological signal processes. However, less information is known about the 14-3-3 gene family in banana. In this study, we performed genome-wide identification and molecular characterization of the 14-3-3 gene family during development, ripening, and response to cold, salt, and osmotic stresses in banana.

### Identification and evolutionary analysis of banana 14-3-3 genes

In this study, genome-wide analysis identified 25 14-3-3 protein genes in the DH-Pahang (*Musa acuminate*, A-genome, 2*n* = 22) genome database. The finding indicated that the banana 14-3-3 gene family had expanded compared to that in *Arabidopsis* (Rosenquist et al., 2001; Chevalier et al., 2009) and rice (Chen et al., 2006; Yao et al., 2007). Based on phylogenetic analysis, they are classified as either the ε group or the non-ε group, which is in accordance with previous phylogenic classification of 14-3-3s in rice (Chen et al., 2006), *Arabidopsis* (Chevalier et al., 2009), soybean (Li and Dhaubhadel, 2011), cotton (Sun et al., 2011), common bean (Tian et al., 2015), *Populus* (Li et al., 2015), *Medicago truncatula* (Qin et al., 2016), and *B. rapa* (Chandna et al., 2016). Gene structure analysis indicated that the number of exons and introns of ε group 14-3-3 genes from banana is more than non-ε group genes (Figure 3) which was also found in *Arabidopsis*, rice, common bean (Tian et al., 2015), *M. truncatula* (Qin et al., 2016), and *B. rapa* (Chandna et al., 2016). Thus, the exon numbers of 14-3-3 genes in each group among 6 species supports their evolutionary classification. Additionally, the exons number of most 14-3-3 genes (except for *MaGRF14*) from banana (2–7) and rice (4–6) is more than that from *Arabidopsis* (3–6), implying that the banana 14-3-3 genes changed greatly during evolution. Conserved motif analysis showed that all identified banana 14-3-3s had the typical 14-3-3 domain. Moreover, the ε group and non-ε group banana 14-3-3s had special signature motifs, respectively, further supporting the classification of 14-3-3s. The conservation and divergence in the protein sequence might be an evolutionary consequence. Together, the identification and classification of the banana 14-3-3 gene family was supported by evolutionary, genetic structure, and conserved motif analyses.

### Involvement of 14-3-3 genes in fruit development, postharvest ripening, and abiotic stress response of banana

Banana fruit quality and shelf life are determined by fruit development and the postharvest ripening process. Thus, it is essential to understand the mechanisms involved in banana fruit development and postharvest ripening. In this study, we studied the expression profiles of banana 14-3-3s in fruit development and postharvest ripening processes. Results indicated that most banana 14-3-3s showed great expression changes. Moreover, many genes showed robust transcriptional abundance (value>30) during the developmental and ripening stages (Figure 6, Table S4). Much evidence had demonstrated that plant 14-3-3 proteins could be involved in fruit development and ripening processes. The distinct transcript accumulation of 2 tomato 14-3-3 homologs suggested their potential involvement in fruit development (Laughner et al., 1995). The *Pp14-3-3a* gene was involved in regulating fruit development (Shi and Zhang, 2014). Expression of mango 14-3-3 genes increased during fruit development and decreased during fruit ripening (Pandit et al., 2010). In *Arabidopsis*, expression of *GF14 chi* was exclusively detected in mature siliques and immature seeds (Daugherty et al., 1996). In maize, expression of 2 14-3-3 proteins changed significantly during seed development (Dou et al., 2014). Based on the evidence above, 14-3-3 genes showed potential involvement in regulating banana fruit development and postharvest ripening processes.

Interestingly, the number of 14-3-3 genes with high transcriptional abundance (value>30) at 0 DAF in BX and FJ is greater than at other stages, suggesting that 14-3-3 genes play a crucial role in early banana fruit development (Figure 6, Table S4). Previous evidences have revealed the importance of 14-3-3 genes during early fruit development (Taoka et al., 2011; Niemenak et al., 2015). Expression of a cacao 14-3-3 protein was detected exclusively in fruit tissues, which is in accordance with fruit development (Niemenak et al., 2015). The rice 14-3-3 protein *GF14c* was perfectly demonstrated as intracellular receptors that induce flowering and play a crucial role in early seed development (Taoka et al., 2011). Overall, these results suggested the key roles of 14-3-3 genes in regulating early fruit development. During the postharvest ripening processes, numerous physiological, biochemical, and molecular changes occur (Roy Choudhury et al., 2009; Shiga et al., 2011; Moser et al., 2012; Etienne et al., 2015) which influence the quality of banana fruit. Thus, postharvest ripening is critical to improving fruit quality and extending fruit shelf life in banana. Notably, 9 genes (*MaGRF1, 5, 6, 9, 10, 13, 15, 19*, and *24*) had higher expression levels at postharvest ripening stages in BX compared to those in FJ, indicating a significant response at the transcriptional levels in the postharvest ripening of BX (Figure 6, Table S4). BX is a more widely cultivated strain than FJ in tropical and subtropical areas because of its high fruit quality (Cruz-Cárdenas et al., 2015). Plant 14-3-3 proteins have been extensively demonstrated as key regulator of primary metabolism and can interact with a wide range of metabolic enzymes (Lu et al., 2016). The robust expression of these *MaGRFs* in BX during the banana fruit ripening process implies their potential involvement in mediating primary metabolism and formation of banana fruit quality.

Increasing evidences have suggested that 14-3-3 genes could transcriptionally respond to abiotic stress in many species (Chen et al., 2006; Xu and Shi, 2006; Sun et al., 2011; Li et al., 2015; Tian et al., 2015; Chandna et al., 2016). Further biochemical and genetic evidences have demonstrated that plant 14-3-3 proteins, acting as signal moderators, positively regulate plants responseto abiotic stress (Campo et al., 2012; He et al., 2015). In this study, many 14-3-3 genes showed great changes after osmotic, cold, and salt treatments in two banana varieties, suggested their potential role in regulating banana response to abiotic stress. Additionally, we noticed that the number of upregulated 14-3-3 genes was greater in BX than in FJ after salt, cold, and osmotic treatments (Figure 7, Table S5). The more upregulated 14-3-3 genes in BX suggested the strong signaling responses of BX plants to abiotic stresses. It is known that BX is sensitive to abiotic stresses relative to FJ (Ravi et al., 2013). Thus, compared with FJ, BX needed to strongly activate 14-3-3 proteins-mediated signaling pathways to combat abiotic stresses. Collectively, it is concluded that 14-3-3 genes might play an essential role in regulating resistance to environmental stresses in banana. These findings established a solid foundation for further studies of the 14-3-3 protein-mediated abiotic stresses signal response in banana.

In conclusion, this study identified 25 banana 14-3-3 genes and established the classification and evolutionary relationship of these genes using phylogenetic, gene structure, and conserved protein motif analyses. Further expression analyses demonstrated that banana 14-3-3 genes were involved in regulating fruit development, postharvest ripening and abiotic stress responses. These data bring new insight to the control of 14-3-3 gene expression, which provides new clues for further functional characterization of potential targets of 14-3-3 and the genetic improvement of bananas.

## Author contributions

AG, ZJ conceived the study. WH, BX, XY, QX, PH, and SX performed the experiments and carried out the analysis. ML, LR, and WH designed the experiments and wrote the manuscript. All authors read and approved the final manuscript.

### Conflict of interest statement

The authors declare that the research was conducted in the absence of any commercial or financial relationships that could be construed as a potential conflict of interest.

## Supplementary material

Table S1 **Amino acid sequence identity of banana 14-3-3 genes**.

Table S2 **Characteristics of 14-3-3s proteins in banana**.

Table S3 **Expression data of the banana 14-3-3 genes in different tissues of BX and FJ**.

Table S4 **Expression data of the banana 14-3-3 genes in different stages of fruit development and ripening in BX and FJ**.

Table S5 **Expression data (log2-based values) of the banana 14-3-3 genes after various abiotic stress treatment in BX and FJ**.

Table S6 **The primer sequences used for qRT-PCR**.

Figure S1 **Conserved amino acid motifs and annotation of 14-3-3s**.

Figure S2 **Expression analysis of MaGRF genes in BX and FJ by qRT-PCR. (A–C)** expression of MaGRF3, MaGRF16, and MaGRF25 in various organs of BX and FJ; **(D–F)** expression of MaGRF1, MaGRF3, and MaGRF25 in distinct stages of fruit development and postharvest ripening in BX and FJ; **(G–I)** expression of MaGRF3, MaGRF16, and MaGRF25 responding to salt, cold, and osmotic stresses in BX and FJ, Log2-based value were used to display differential expression results. Data are means ± *SD* of *n* = 3 replicates.

### Availability of supporting data

The sequences of DH-Pahang 14-3-3 genes identified in this study could be available in banana genome database (http://banana-genome.cirad.fr/; The accession numbers were listed in Table S2).

## References

[B1] Bunney T. D. van den Wijngaard P. W. de Boer A. H. (2002). 14-3-3 protein regulation of proton pumps and ion channels. Plant Mol. Biol. 50, 1041–1051. 10.1023/A:1021231805697 12516871

[B2] Campo S. Peris-Peris C. Montesinos L. Peñas G. Messeguer J. San Segundo B. (2012). Expression of the maize ZmGF14-6 gene in rice confers tolerance to drought stress while enhancing susceptibility to pathogen infection. J. Exp. Bot. 63, 983–999. 10.1093/jxb/err328 22016430 PMC3254693

[B3] Catalá R. López-Cobollo R. Mar Castellano M. Angosto T. Alonso J. M. Ecker J. R.. (2014). The *Arabidopsis* 14-3-3 protein RARE COLD INDUCIBLE 1A links low-temperature response and ethylene biosynthesis to regulate freezing tolerance and cold acclimation. Plant Cell 26, 3326–3342. 10.1105/tpc.114.127605 25122152 PMC4371832

[B4] Chandna R. Augustine R. Kanchupati P. Kumar R. Kumar P. Arya G. C.. (2016). Class-specific evolution and transcriptional differentiation of 14-3-3 family members in mesohexaploid *Brassica rapa*. Front. Plant Sci. 7:12. 10.3389/fpls.2016.00012 26858736 PMC4726770

[B5] Chen F. Li Q. Sun L. He Z. (2006). The rice 14-3-3 gene family and its involvement in responses to biotic and abiotic stress. DNA Res. 13, 53–63. 10.1093/dnares/dsl001 16766513

[B6] Chen L. Zhong H. Y. Kuang J. F. Li J. G. Lu W. J. Chen J. Y. (2011). Validation of reference genes for RT-qPCR studies of gene expression in banana fruit under different experimental conditions. Planta 234, 377–390. 10.1007/s00425-011-1410-3 21505864

[B7] Chen Q. Kan Q. Wang P. Yu W. Yu Y. Zhao Y.. (2015). Phosphorylation and interaction with the 14-3-3 protein of the plasma membrane H^+^-ATPase are involved in the regulation of magnesium-mediated increases in aluminum-induced citrate exudation in broad bean (*Vicia faba*. L). Plant Cell Physiol. 56, 1144–1153. 10.1093/pcp/pcv038 25745032

[B8] Chevalier D. Morris E. R. Walker J. C. (2009). 14-3-3 and FHA domains mediate phosphoprotein interactions. Annu. Rev. Plant Biol. 60, 67–91. 10.1146/annurev.arplant.59.032607.092844 19575580

[B9] Comparot S. Lingiah G. Martin T. (2003). Function and specificity of 14-3-3 proteins in the regulation of carbohydrate and nitrogen metabolism. J. Exp. Bot. 54, 595–604. 10.1093/jxb/erg057 12508070

[B10] Cotelle V. Leonhardt N. (2016). 14-3-3 proteins in guard cell signaling. Front Plant Sci. 6:1210. 10.3389/fpls.2015.01210 26858725 PMC4729941

[B11] Cruz-Cárdenas C. I. Miranda-Ham M. L. Castro-Concha L. A. Ku-Cauich J. R. Vergauwen R. Reijnders T.. (2015). Fructans and other water soluble carbohydrates in vegetative organs and fruits of different *Musa* spp. accessions. Front Plant Sci. 6:395. 10.3389/fpls.2015.00395 26106398 PMC4460310

[B12] Daugherty C. J. Rooney M. F. Miller P. W. Ferl R. J. (1996). Molecular organization and tissue-specific expression of an *Arabidopsis* 14-3-3 gene. Plant Cell 8, 1239–1248. 10.1105/tpc.8.8.1239 8776894 PMC161235

[B13] de Boer A. H. van Kleeff P. J. Gao J. (2013). Plant 14-3-3 proteins as spiders in a web of phosphorylation. Protoplasma 250, 425–440. 10.1007/s00709-012-0437-z 22926776

[B14] DeLille J. M. Sehnke P. C. Ferl R. J. (2001). The *Arabidopsis* 14-3-3 family of signaling regulators. Plant Physiol. 126, 35–38. 10.1104/pp.126.1.35 11351068 PMC1540106

[B15] de Vetten N. C. Lu G. Feri R. J. (1992). A maize protein associated with the G-box binding complex has homology to brain regulatory proteins. Plant Cell 4, 1295–1307. 10.1105/tpc.4.10.1295 1446170 PMC160216

[B16] D'Hont A. Denoeud F. Aury J. M. Baurens F. C. Carreel F. Garsmeur O.. (2012). The banana (*Musa acuminata*) genome and the evolution of monocotyledonous plants. Nature 488, 213–217. 10.1038/nature11241 22801500

[B17] Diaz C. Kusano M. Sulpice R. Araki M. Redestig H. Saito K.. (2011). Determining novel functions of *Arabidopsis* 14-3-3 proteins in central metabolic processes. BMC Syst. Biol. 5:192. 10.1186/1752-0509-5-192 22104211 PMC3253775

[B18] Dou Y. Liu X. Yin Y. Han S. Lu Y. Liu Y.. (2014). Affinity chromatography revealed insights into unique functionality of two 14-3-3 protein species in developing maize kernels. J. Proteomics 114, 274–286. 10.1016/j.jprot.2014.10.019 25449830

[B19] Etienne A. Génard M. Bugaud C. (2015). A process-based model of TCA cycle functioning to analyze citrate accumulation in pre- and post-harvest fruits. PLoS ONE 10:e0126777. 10.1371/journal.pone.0126777 26042830 PMC4456289

[B20] Finn R. D. Clements J. Eddy S. R. (2011). HMMER web server: interactive sequence similarity searching. Nucleic Acids Res. 39, 29–37. 10.1093/nar/gkr367 21593126 PMC3125773

[B21] He Y. Wu J. Lv B. Li J. Gao Z. Xu W.. (2015). Involvement of 14-3-3 protein GRF9 in root growth and response under polyethylene glycol-induced water stress. J. Exp. Bot. 66, 2271–2281. 10.1093/jxb/erv149 25873671 PMC4986726

[B22] Ho S. L. Huang L. F. Lu C. A. He S. L. Wang C. C. Yu S. P.. (2013). Sugar starvation- and GA-inducible calcium-dependent protein kinase 1 feedback regulates GA biosynthesis and activates a 14-3-3 protein to confer drought tolerance in rice seedlings. Plant Mol. Biol. 81, 347–361. 10.1007/s11103-012-0006-z 23329372

[B23] Ito T. Nakata M. Fukazawa J. Ishida S. Takahashi Y. (2014). Scaffold function of Ca^2+^-dependent protein kinase: tobacco Ca^2+^-DEPENDENT PROTEIN KINASE1 transfers 14-3-3 to the substrate REPRESSION OF SHOOT GROWTH after phosphorylation. Plant Physiol. 165, 1737–1750. 10.1104/pp.114.236448 24920444 PMC4119052

[B24] Jaspert N. Throm C. Oecking C. (2011). *Arabidopsis* 14-3-3 proteins: fascinating and less fascinating aspects. Front. Plant Sci. 2:96. 10.3389/fpls.2011.00096 22639620 PMC3355631

[B25] Kanczewska J. Marco S. Vandermeeren C. Maudoux O. Rigaud J. L. Boutry M. (2005). Activation of the plant plasma membrane H^+^-ATPase by phosphorylation and binding of 14-3-3 proteins converts a dimer into a hexamer. Proc. Natl. Acad. Sci. U.S.A. 102, 11675–11680. 10.1073/pnas.0504498102 16081536 PMC1187987

[B26] Kawamoto N. Sasabe M. Endo M. Machida Y. Araki T. (2015). Calcium-dependent protein kinases responsible for the phosphorylation of a bZIP transcription factor FD crucial for the florigen complex formation. Sci. Rep. 5:8341. 10.1038/srep08341 25661797 PMC4321167

[B27] Kumar K. Muthamilarasan M. Bonthala V. S. Roy R. Prasad M. (2015). Unraveling 14-3-3 proteins in C4 panicoids with emphasis on model plant *Setaria italica* reveals phosphorylation-dependent subcellular localization of RS splicing factor. PLoS ONE 10:e0122536. 10.1371/journal.pone.0123236 25849294 PMC4388342

[B28] Larkin M. A. Blackshields G. Brown N. P. Chenna R. McGettigan P. A. McWilliam H.. (2007). Clustal, W., and Clustal X version 2.0. Bioinformatics 23, 2947–2948. 10.1093/bioinformatics/btm404 17846036

[B29] Latz A. Mehlmer N. Zapf S. Mueller T. D. Wurzinger B. Pfister B.. (2013). Salt stress triggers phosphorylation of the *Arabidopsis* vacuolar K^+^ channel TPK1 by calcium-dependent protein kinases (CDPKs). Mol. Plant 6, 1274–1289. 10.1093/mp/sss158 23253603 PMC3971370

[B30] Laughner B. Lawrence S. D. Ferl R. J. (1995). Two cDNA clones encoding 14-3-3 homologs from tomato fruit. Biochim. Biophys. Acta 11263, 67–70. 10.1016/0167-4781(95)00092-U 7632735

[B31] Li R. Jiang X. Jin D. Dhaubhadel S. Bian S. Li X. (2015). Identification of 14-3-3 family in common bean and their response to abiotic stress. PLoS ONE 10:e0142580. 10.1371/journal.pone.0143280 26599110 PMC4658069

[B32] Li X. Dhaubhadel S. (2011). Soybean 14-3-3 gene family: identification and molecular characterization. Planta 233, 569–582. 10.1007/s00425-010-1315-6 21120521

[B33] Lu Y. Yasuda S. Li X. Fukao Y. Tohge T. Fernie A. R.. (2016). Characterization of ubiquitin ligase SlATL31 and proteomic analysis of 14-3-3 targets in tomato fruit tissue (*Solanum lycopersicum* L.). J. Proteomics 143, 254–264. 10.1016/j.jprot.2016.04.016 27113132

[B34] Moser S. Müller T. Holzinger A. Lütz C. Kräutler B. (2012). Structures of chlorophyll catabolites in bananas (*Musa acuminata*) reveal a split path of chlorophyll breakdown in a ripening fruit. Chemistry 18, 10873–10885. 10.1002/chem.201201023 22807397 PMC3499688

[B35] Niemenak N. Kaiser E. Maximova S. N. Laremore T. Guiltinan M. J. (2015). Proteome analysis during pod, zygotic and somatic embryo maturation of *Theobroma cacao*. J. Plant Physiol. 180, 49–60. 10.1016/j.jplph.2015.02.011 25889873

[B36] Ottmann C. Marco S. Jaspert N. Marcon C. Schauer N. Weyand M.. (2007). Structure of a 14-3-3 coordinated hexamer of the plant plasma membrane H^+^-ATPase by combining X-ray crystallography and electron cryomicroscopy. Mol Cell 25, 427–440. 10.1016/j.molcel.2006.12.017 17289589

[B37] Pandit S. S. Kulkarni R. S. Giri A. P. Köllner T. G. Degenhardt J. Gershenzon J.. (2010). Expression profiling of various genes during the fruit development and ripening of mango. Plant Physiol. Biochem. 48, 426–433. 10.1016/j.plaphy.2010.02.012 20363641

[B38] Paul A. L. Denison F. C. Schultz E. R. Zupanska A. K. Ferl R. J. (2012). 14-3-3 phosphoprotein interaction networks - does isoform diversity present functional interaction specification? Front. Plant Sci. 3:190. 10.3389/fpls.2012.00190 22934100 PMC3422896

[B39] Pua E. C. Chandramouli S. Han P. Liu P. (2003). Malate synthase gene expression during fruit ripening of Cavendish banana (*Musa acuminata* cv. Williams). J. Exp. Bot. 54, 309–316. 10.1093/jxb/erg030 12493858

[B40] Qin C. Cheng L. Shen J. Zhang Y. Cao H. Lu D.. (2016). Genome-wide identification and expression analysis of the 14-3-3 family genes in *Medicago truncatula*. Front Plant Sci. 7:320. 10.3389/fpls.2016.00320 27047505 PMC4801894

[B41] Ravi I. Uma S. Vaganan M. M. Mustaffa M. M. (2013). Phenotyping bananas for drought resistance. Front. Physiol. 4:9. 10.3389/fphys.2013.00009 23443573 PMC3580962

[B42] Raza W. Ling N. Zhang R. Huang Q. Xu Y. Shen Q. (2016). Success evaluation of the biological control of Fusarium wilts of cucumber, banana, and tomato since 2000 and future research strategies. Crit. Rev. Biotechnol. 26, 1–11. 10.3109/07388551.2015.1130683 26810104

[B43] Rosenquist M. Alsterfjord M. Larsson C. Sommarin M. (2001). Data mining the *Arabidopsis* genome reveals fifteen 14-3-3 genes. Expression is demonstrated for two out of five novel genes. Plant Physiol. 127, 142–149. 10.1104/pp.127.1.142 11553742 PMC117970

[B44] Roy Choudhury S. Roy S. Sengupta D. N. (2009). Characterization of cultivar differences in beta-1, 3 glucanase gene expression, glucanase activity and fruit pulp softening rates during fruit ripening in three naturally occurring banana cultivars. Plant Cell Rep. 28, 1641–1653. 10.1007/s00299-009-0764-5 19697038

[B45] Schoonheim P. J. Sinnige M. P. Casaretto J. A. Veiga H. Bunney T. D. Quatrano R. S.. (2007). 14-3-3 adaptor proteins are intermediates in ABA signal transduction during barley seed germination. Plant J. 49, 289–301. 10.1111/j.1365-313X.2006.02955.x 17241451

[B46] Sehnke P. C. DeLille J. M. Ferl R. J. (2002). Consummating signal transduction: the role of 14-3-3 proteins in the completion of signal-induced transitions in protein activity. Plant Cell 14(Suppl.), S339–S354. 10.1105/tpc.010430 12045287 PMC151265

[B47] Shi H. Zhang Y. (2014). Pear 14-3-3a gene (Pp14-3-3a) is regulated during fruit ripening and senescense, and involved in response to salicylic acid and ethylene signalling. J. Genet. 93, 747–753. 10.1007/s12041-014-0447-z 25572233

[B48] Shiga T. M. Soares C. A. Nascimento J. R. Purgatto E. Lajolo F. M. Cordenunsi B. R. (2011). Ripening-associated changes in the amounts of starch and non-starch polysaccharides and their contributions to fruit softening in three banana cultivars. J. Sci. Food Agric. 91, 1511–1516. 10.1002/jsfa.4342 21445854

[B49] Sun G. Xie F. Zhang B. (2011). Transcriptome-wide identification and stress properties of the 14-3-3 gene family in cotton (*Gossypium hirsutum* L.). Funct. Integr. Genomics. 11, 627–636. 10.1007/s10142-011-0242-3 21805362

[B50] Sun X. Luo X. Sun M. Chen C. Ding X. Wang X.. (2014). A Glycine soja 14-3-3 protein GsGF14o participates in stomatal and root hair development and drought tolerance in *Arabidopsis thaliana*. Plant Cell Physiol. 55, 99–118. 10.1093/pcp/pct161 24272249

[B51] Tamura K. Peterson D. Peterson N. Stecher G. Nei M. Kumar S. (2011). MEGA5: molecular evolutionary genetics analysis using maximum likelihood, evolutionary distance, and maximum parsimony methods. Mol. Biol. Evol. 28, 2731–2739. 10.1093/molbev/msr121 21546353 PMC3203626

[B52] Taoka K. Ohki I. Tsuji H. Furuita K. Hayashi K. Yanase T.. (2011). 14-3-3 proteins act as intracellular receptors for rice Hd3a florigen. Nature 476, 332–335. 10.1038/nature10272 21804566

[B53] Tian F. Wang T. Xie Y. Zhang J. Hu J. (2015). Genome-wide identification, classification, and expression analysis of 14-3-3 gene family in Populus. PLoS ONE 10:e0122525. 10.1371/journal.pone.0123225 25867623 PMC4395111

[B54] Tseng T. S. Whippo C. Hangarter R. P. Briggs W. R. (2012). The role of a 14-3-3 protein in stomatal opening mediated by PHOT2 in *Arabidopsis*. Plant Cell 24, 1114–1126. 10.1105/tpc.111.092130 22408078 PMC3336120

[B55] van Kleeff P. J. Jaspert N. Li K. W. Rauch S. Oecking C. de Boer A. H. (2014). Higher order *Arabidopsis* 14-3-3 mutants show 14-3-3 involvement in primary root growth both under control and abiotic stress conditions. J. Exp. Bot. 65, 5877–5888. 10.1093/jxb/eru338 25189593 PMC4203132

[B56] Vercruyssen L. Tognetti V. B. Gonzalez N. Van Dingenen J. De Milde L. Bielach A.. (2015). GROWTH REGULATING FACTOR5 stimulates Arabidopsis chloroplast division, photosynthesis, and leaf longevity. Plant Physiol. 167, 817–825. 10.1104/pp.114.256180 25604530 PMC4348790

[B57] Xu W. F. Shi W. M. (2006). Expression profiling of the 14-3-3 gene family in response to salt stress and potassium and iron deficiencies in young tomato (*Solanum lycopersicum*) roots: analysis by real-time RT-PCR. Ann. Bot. 98, 965–974. 10.1093/aob/mcl189 16943217 PMC2803592

[B58] Yan J. He C. Wang J. Mao Z. Holaday S. A. Allen R. D.. (2004). Overexpression of the Arabidopsis 14-3-3 protein GF14 lambda in cotton leads to a “stay-green” phenotype and improves stress tolerance under moderate drought conditions. Plant Cell Physiol. 45, 1007–1014. 10.1093/pcp/pch115 15356326

[B59] Yan Y. Takáč T. Li X. Chen H. Wang Y. Xu E.. (2015). Variable content and distribution of arabinogalactan proteins in banana (*Musa* spp.) under low temperature stress. Front. Plant Sci. 6:353. 10.3389/fpls.2015.00353 26074928 PMC4444754

[B60] Yang Q. S. Gao J. He W. D. Dou T. X. Ding L. J. Wu J. H.. (2015). Comparative transcriptomics analysis reveals difference of key gene expression between banana and plantain in response to cold stress. BMC Genomics 16:446. 10.1186/s12864-015-1551-z 26059100 PMC4461995

[B61] Yao Y. Du Y. Jiang L. Liu J. Y. (2007). Molecular analysis and expression patterns of the 14-3-3 gene family from *Oryza sativa*. J. Biochem. Mol. Biol. 40, 349–357. 10.5483/bmbrep.2007.40.3.349 17562286

[B62] Zhou H. Lin H. Chen S. Becker K. Yang Y. Zhao J.. (2014). Inhibition of the *Arabidopsis* salt overly sensitive pathway by 14-3-3 proteins. Plant Cell 26, 1166–1182. 10.1105/tpc.113.117069 24659330 PMC4001376

[B63] Zhou Y. Zhang Z. T. Li M. Wei X. Z. Li X. J. Li B. Y.. (2015). Cotton (*Gossypium hirsutum*) 14-3-3 proteins participate in regulation of fibre initiation and elongation by modulating brassinosteroid signalling. Plant Biotechnol. J. 13, 269–280. 10.1111/pbi.12275 25370928

