# Supplementary figures and images for: Genome-Wide Identification, Phylogeny, and Expression Analyses of the 14-3-3 Family Reveal Their Involvement in the Development, Ripening, and Abiotic Stress Response in Banana
Source: Front Plant Sci. 2016 Sep 22;7:1442. doi: 10.3389/fpls.2016.01442 (PMC5031707; doi:10.3389/fpls.2016.01442)

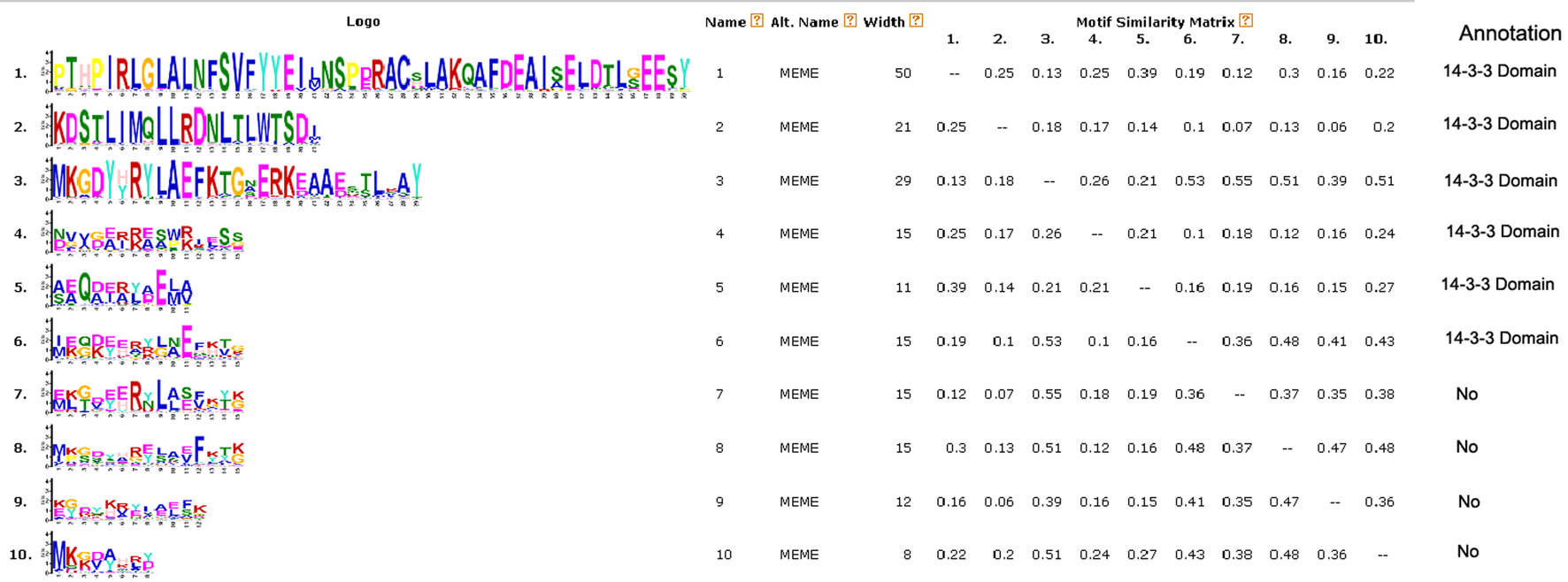

Supplement: Figure S1 — Conserved amino acid motifs and annotation of 14-3-3s. [file Image1.TIF]

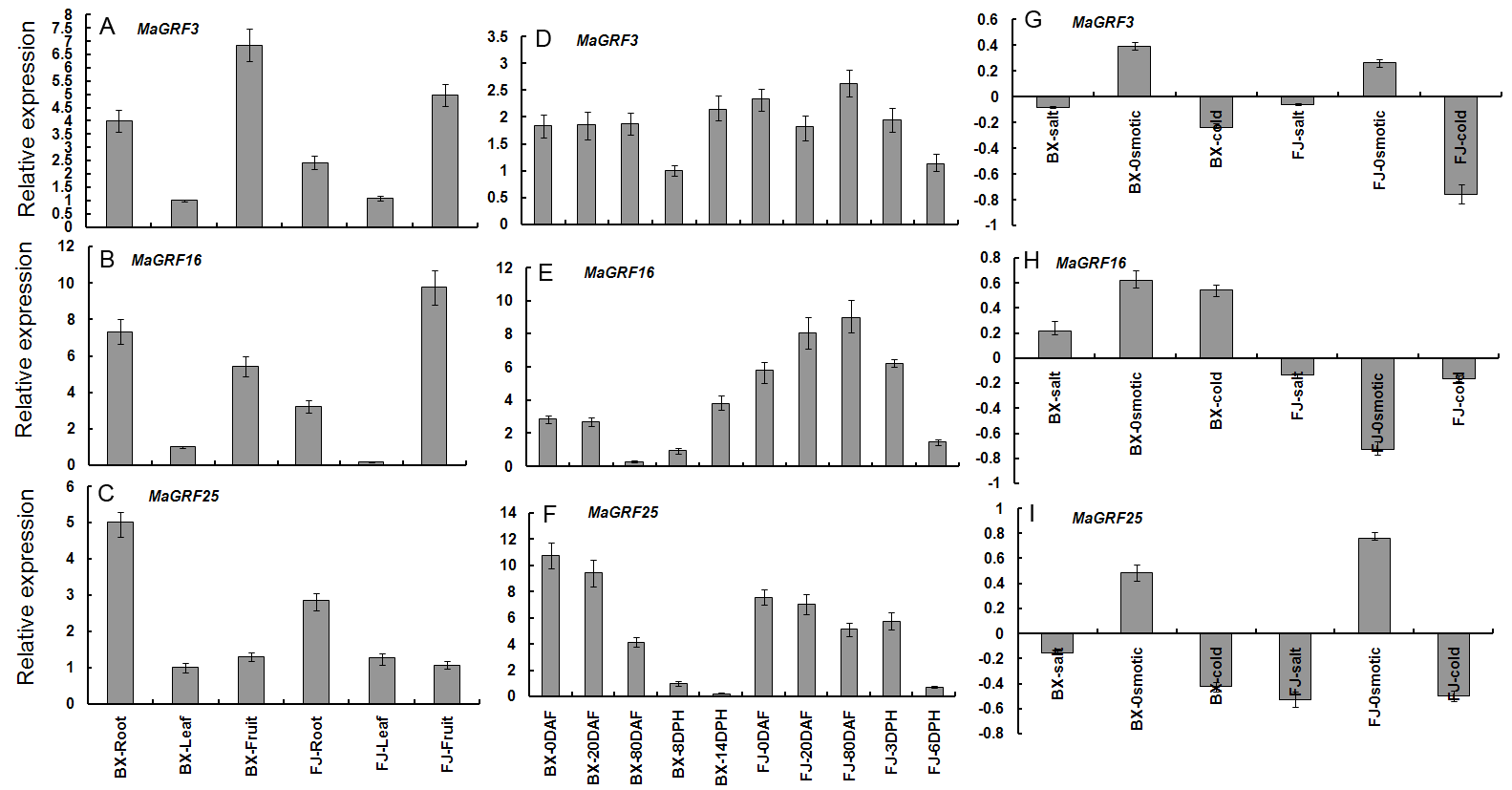

Supplement: Figure S2 — Expression analysis of MaGRF genes in BX and FJ by qRT-PCR. (A–C) expression of MaGRF3, MaGRF16, and MaGRF25 in various organs of BX and FJ; (D–F) expression of MaGRF1, MaGRF3, and MaGRF25 in distinct stages of fruit development and postharvest ripening in BX and FJ; (G–I) expression of MaGRF3, MaGRF16, and MaGRF25 responding to salt, cold, and osmotic stresses in BX and FJ, Log2-based value were used to display differential expression results. Data are means ± SD of n = 3 replicates. [file Image2.TIF]
